# Management of Severe SARS-CoV-2-Associated Organizing Pneumonia With Immunoglobulins

**DOI:** 10.7759/cureus.77120

**Published:** 2025-01-08

**Authors:** Bimaje Akpa

**Affiliations:** 1 Pulmonary and Critical Care Medicine, University of Minnesota School of Medicine, Minneapolis, USA

**Keywords:** corticosteroids in covid-19, covid- 19, igg deficiency, immunodeficiency, immunoglobulins, interstitial lung disease, organizing pneumonia, post-covid-19 syndrome, sars-cov-2, virus

## Abstract

Organizing pneumonia (OP) is a pulmonary inflammatory disorder involving the alveolar airspaces and ducts and resulting in interstitial lung disease. It is usually corticosteroid responsive. There have been reports of corticosteroid-resistant and refractory cases of organizing pneumonia (OP) requiring alternative treatment options, including immunosuppressants and cytotoxic agents. There are only a handful of cases of organizing pneumonia (OP) treated successfully with intravenous immunoglobulins (IVIg). We describe the novel case of a patient with a history of seronegative rheumatoid arthritis presenting with fever, increasing dyspnea, and diffuse opacities on chest imaging. Bronchoscopy revealed positive SARS-CoV-2 antigen, and she was treated with steroids. Due to recurrence of symptoms and worsening hypoxemia while on prolonged glucocorticoid taper, OP was confirmed on lung biopsy. The patient was placed on mycophenolate and glucocorticosteroids, but clinical and radiologic remission was not achieved. She was found to have immunoglobulin (IgG) deficiency and IVIg treatment was initiated which induced a successful clinical and radiologic response.

This novel case highlights the need to consider IgG immune deficiency state when managing steroid- and immunosuppressant-resistant SARS-CoV-2-associated OP.

## Introduction

Organizational pneumonia (OP), a pulmonary inflammatory disorder, affects the alveolar airspaces and ducts and causes interstitial lung disease [1]. When there is no identifiable cause, it is termed cryptogenic organizing pneumonia (COP) [1-3]. It may result from several causes such as infection (including viruses), drugs, radiation and auto-immune disease and in such cases, it is described as secondary organizing pneumonia [4-6]. Since the beginning of the COVID-19 pandemic, clinical, radiological, and histopathological studies have shown that OP is a possible complication of the SARS-CoV-2 infection [7,8].

OP often presents with respiratory symptoms including cough, fever, shortness of breath, and fatigue; however, there is usually some heterogeneity to its clinical presentation [2]. Physical examination is typical for inspiratory crackles or rales. Radiologic findings include infiltrates or opacities on chest radiographs. Consolidations and ground-glass opacities are usually seen on computed tomography (CT) scans [3,9]. Histopathologic evaluation reveals characteristic findings of small airways, alveolar spaces, and ducts filled with intraluminal plugs of cellular debris in a background of interstitial inflammation involving fibroblasts and foamy macrophages [3,9]. OP is usually responsive to steroids with clinical improvement in more than fifty percent of cases. There have been reports of corticosteroid-resistant and refractory cases of OP requiring alternative treatment options, including immunosuppressants and cytotoxic agents [3,6]. However, when complete clinical and radiologic resolution cannot be achieved even with these alternative treatments, management options become very limited. We report a case of corticosteroid- and immunosuppressant-resistant COVID-19-induced OP requiring treatment with intravenous immunoglobulins (IVIg).

## Case presentation

**Figure 1:**
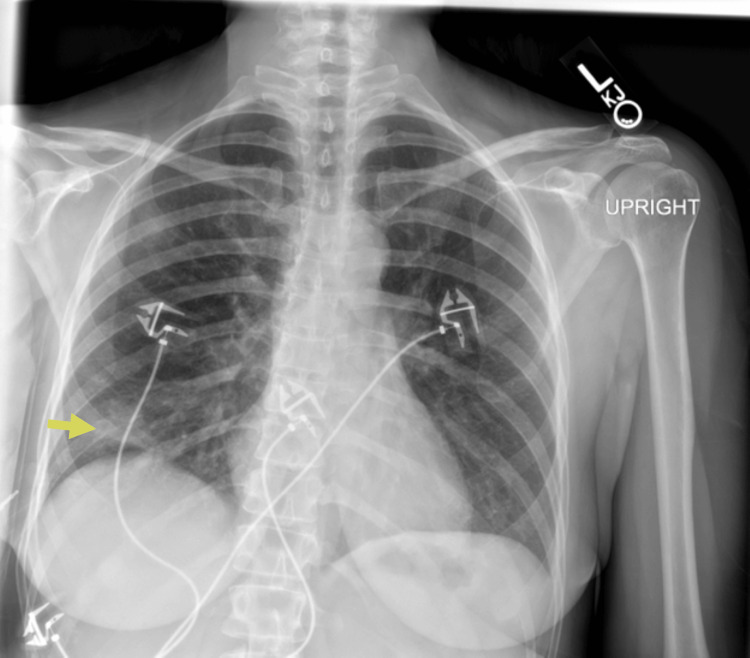
Chest radiograph showing right basilar opacities (yellow arrow)

**Figure 2:**
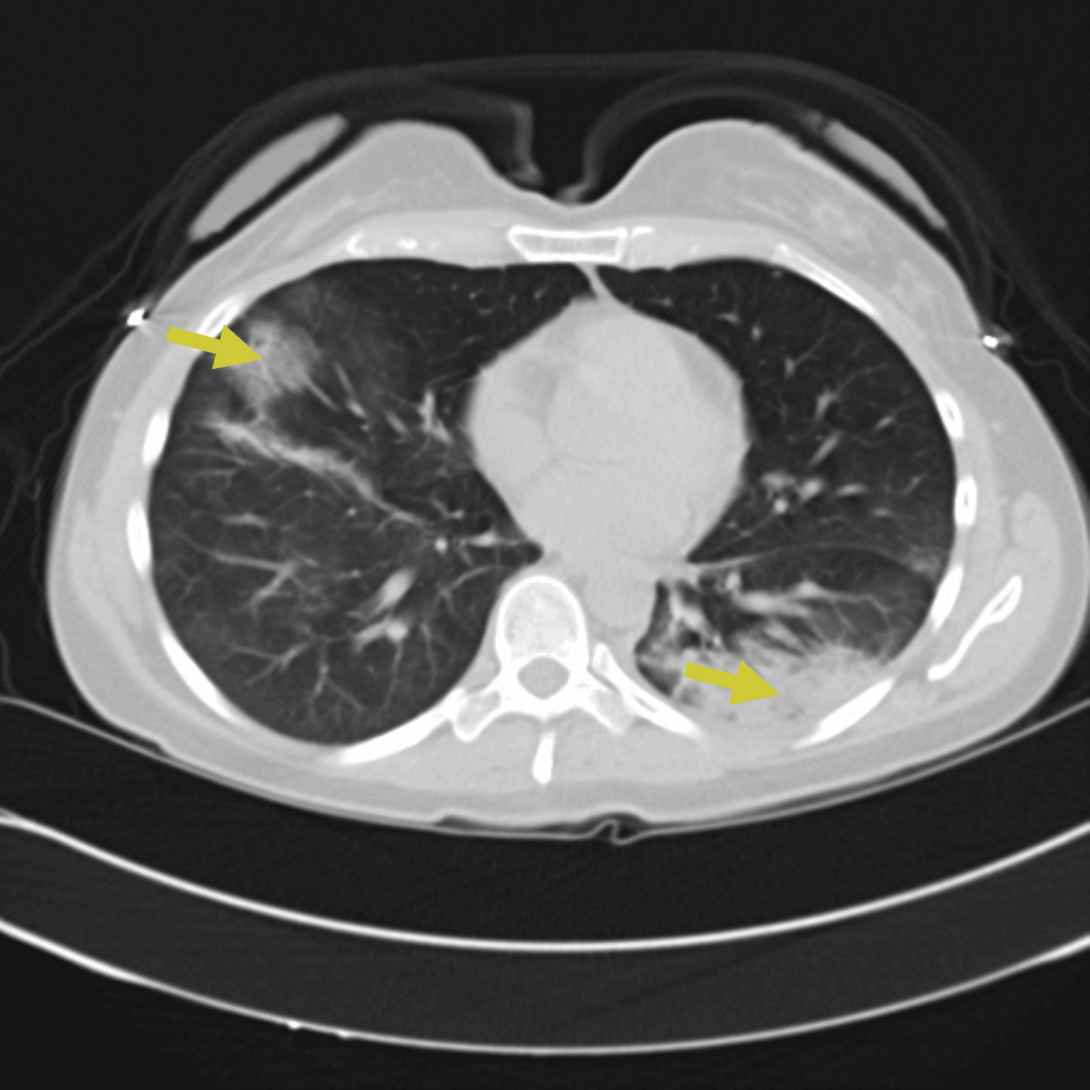
CT chest obtained on presentation showing bilateral ground-glass opacities and consolidations (yellow arrows)

A 45-year-old female with a history of early-stage (stage 1) seronegative rheumatoid arthritis, well controlled on low-dose prednisone, presented to the ED with cough, ongoing fevers, and night sweats. The patient described three days of worsening dyspnea present at rest. She was hospitalized for similar symptoms about six months prior to this index presentation and was diagnosed with SARS-CoV-2 pneumonia based on positive SARS-CoV-2 polymerase chain reaction (PCR) and typical ground-glass infiltrates on computed tomography (CT) scan of the chest at that time. She was treated with prednisone 40 mg orally for seven days with clinical improvement and was eventually discharged. Chest imaging obtained six weeks following discharge at that time showed new pulmonary infiltrates and spontaneous resolution of the previously seen lung findings. She continued to have waxing and waning symptoms of dyspnea on exertion and subjective fevers six months later leading up to this admission and never sought medical care until now. In the ED, the patient appeared comfortable on general examination. She was febrile to 101.4 degrees Fahrenheit and required 2 liters per minute of supplemental oxygen to keep saturations above 90 percent. Initial investigations indicated leukocytosis (16,800 cells/μL with 96% neutrophils), a sedimentation rate of 49 mm/hour (normal < 15 mm/hr), a C-reactive protein level of 4.4 mg/dl (normal 0.02-0.80 mg/dl), procalcitonin < 0.05 ng/ml (normal < 0.05 ng/ml), and D-dimer was not elevated. There was a subtle right lower lobe consolidation on present chest X-ray (Figure 1) and widespread pulmonary infiltrates visible on CT of the chest (Figure 2). Her baseline immunoglobulin panel three months prior to the previous admission is as follows: IgG 824 mg/dl (normal 610-1616 mg/dl), IgA 126 mg/dl (normal 84-499 mg/dl), and IgM 112 mg/dl (normal 35-242 mg/dl).

The patient was admitted for presumed sepsis secondary to multifocal pneumonia and treated with ceftriaxone and azithromycin. 

**Figure 3:**
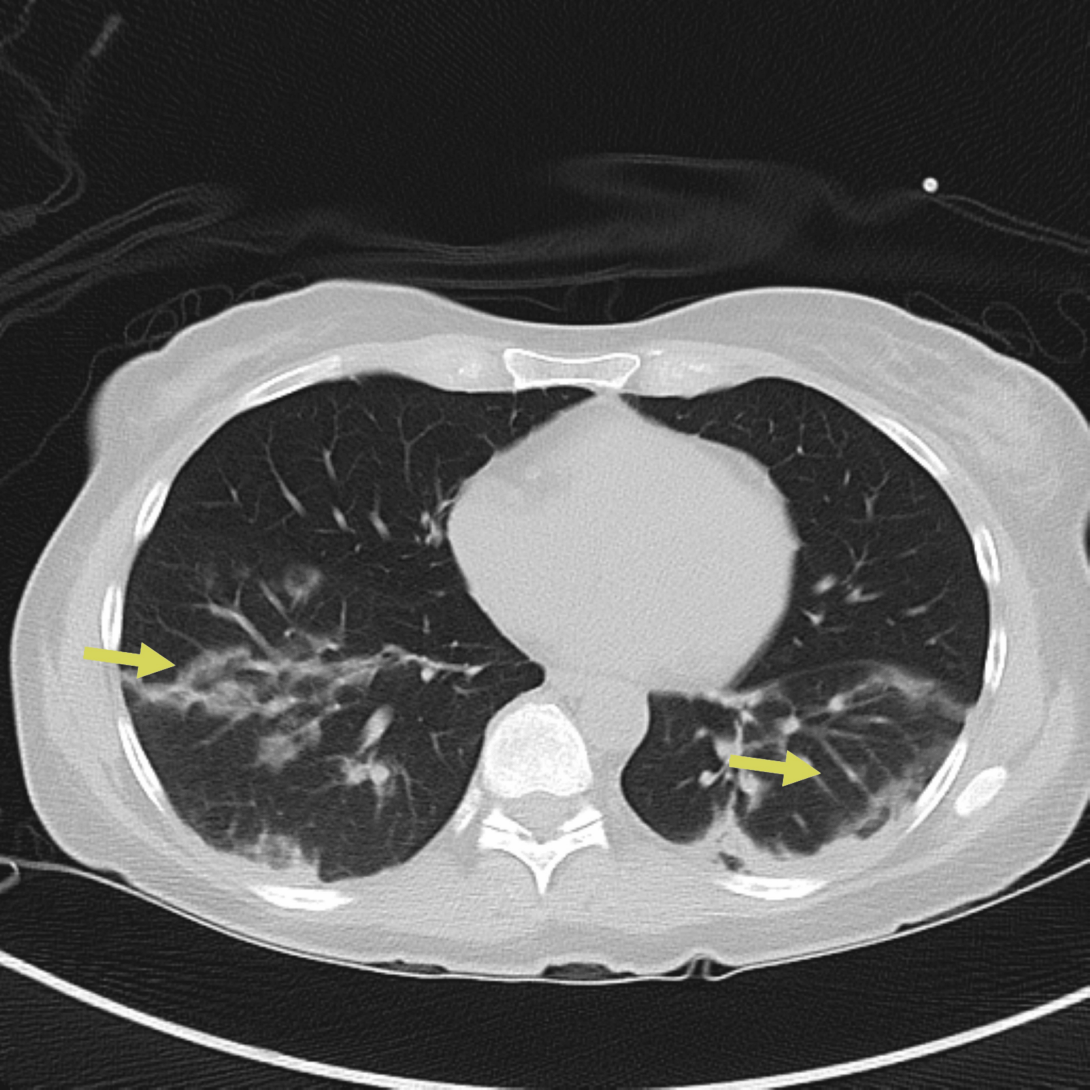
Repeat CT chest on day three of hospitalization showing worsening mixed consolidative and ground-glass opacities, particularly on the left lung (yellow arrows)

Despite antimicrobial therapy, the patient’s condition deteriorated with worsening hypoxemia and increasing supplemental oxygen up to 5 liters per minute of supplemental oxygen to keep saturations above 90 percent. A consultation was placed with the pulmonology and rheumatology service. Three days after admission, a repeat CT scan of the chest demonstrated worsening mixed consolidative and ground-glass opacities (Figure 3).

**Figure 4:**
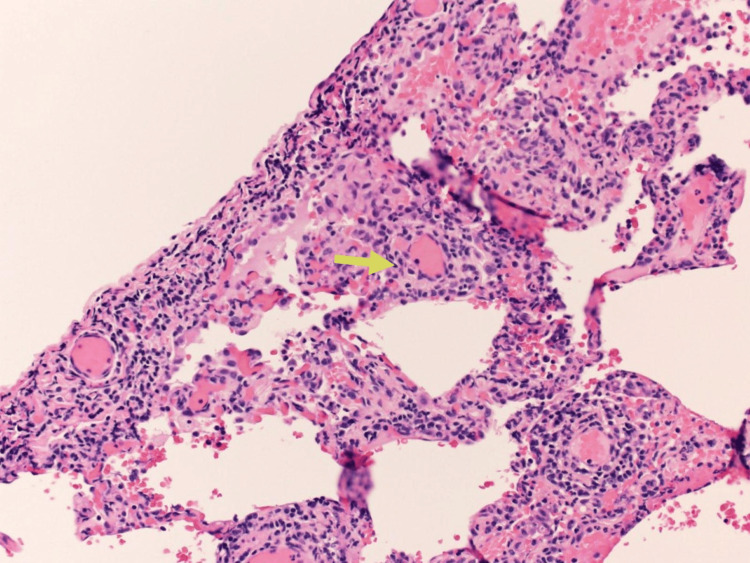
Photomicrograph showing Masson bodies or intraluminal collagen plugs (yellow arrow)

A limited bronchoscopy was performed on day three. Fluid studies from bronchoalveolar lavage (BALF) showed 247 nucleated cells/uL, 12% neutrophils, 9% lymphocytes, 1% monocytes, and no eosinophils noted. Bronchoalveolar lavage studies showed no bacterial, viral, or fungal growth. BALF PCR for pneumocystis jirovecii pneumonia was negative. The patient commenced on IV prednisolone 40 mg/day. On day five, the patient underwent video-assisted thoracoscopic surgery for lung biopsy. The pathology revealed an inflammatory process with foamy macrophages within the alveoli space accompanied by local fibrosis and activated fibroblasts, compatible with organizing pneumonia (Figure 4).

High-dose steroids, 1 mg/kg of body weight methylprednisolone, were started, and the patient made a significant improvement and was weaned off oxygen completely. She was discharged with a prolonged prednisone taper. 

**Figure 5:**
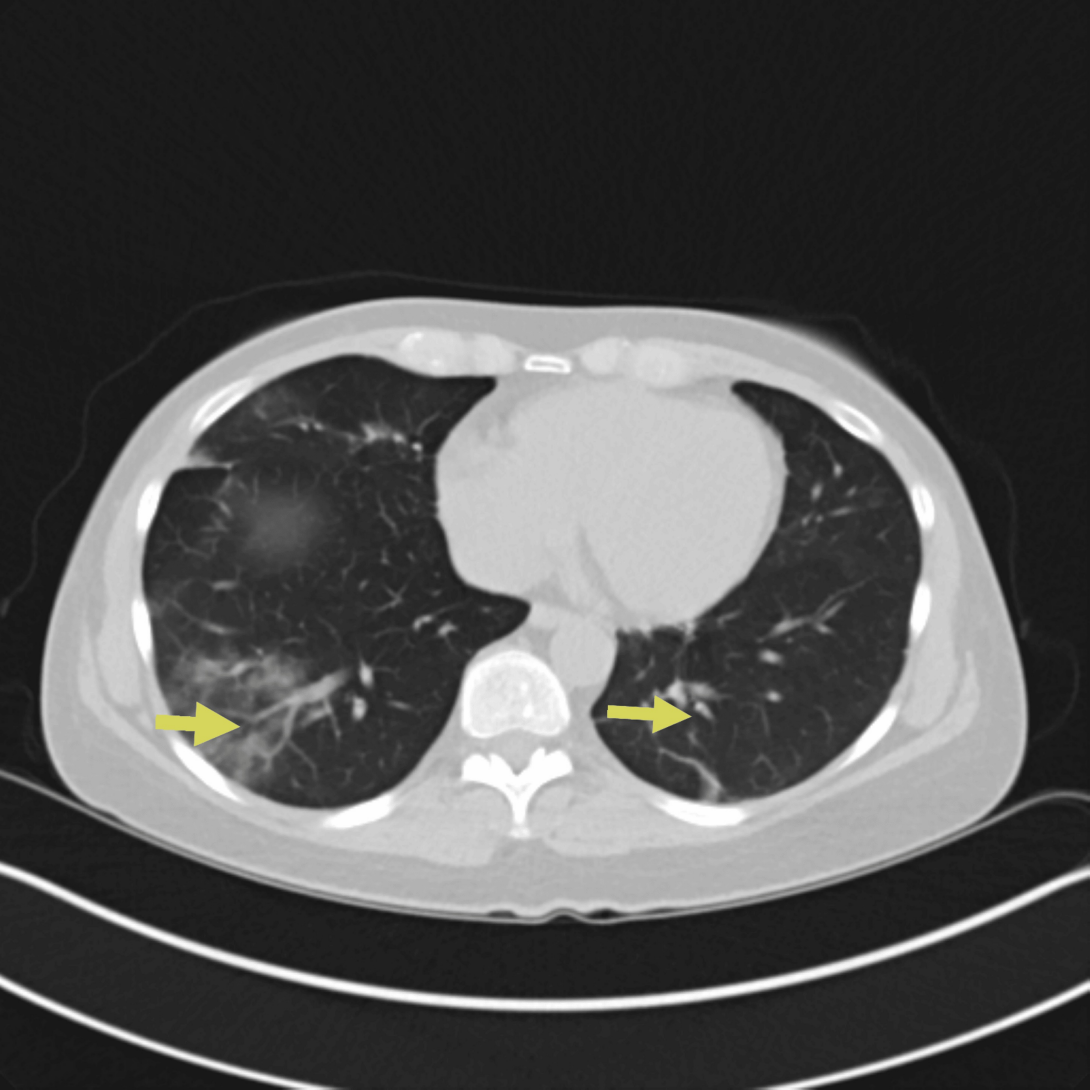
CT chest showing persistent bilateral pulmonary opacities/ground-glass opacities (yellow arrows) The CT chest findings improved compared to Figure 1.

Four weeks after discharge, on subsequent pulmonary outpatient follow-up, it was difficult to wean down her prednisone dose as she continued to have waxing and waning symptoms of dyspnea on exertion and subjective fevers as well as fleeting pulmonary infiltrates (Figure 5). 

Mycophenolate mofetil 500 mg twice daily was initiated at the pulmonary clinic visit with plans for escalating weekly doses to 1000 mg twice daily to help wean down prednisone, but there was no improvement. She appeared to have gastrointestinal (GI) intolerance to mycophenolate and was stopped after three days of starting medication.

**Figure 6:**
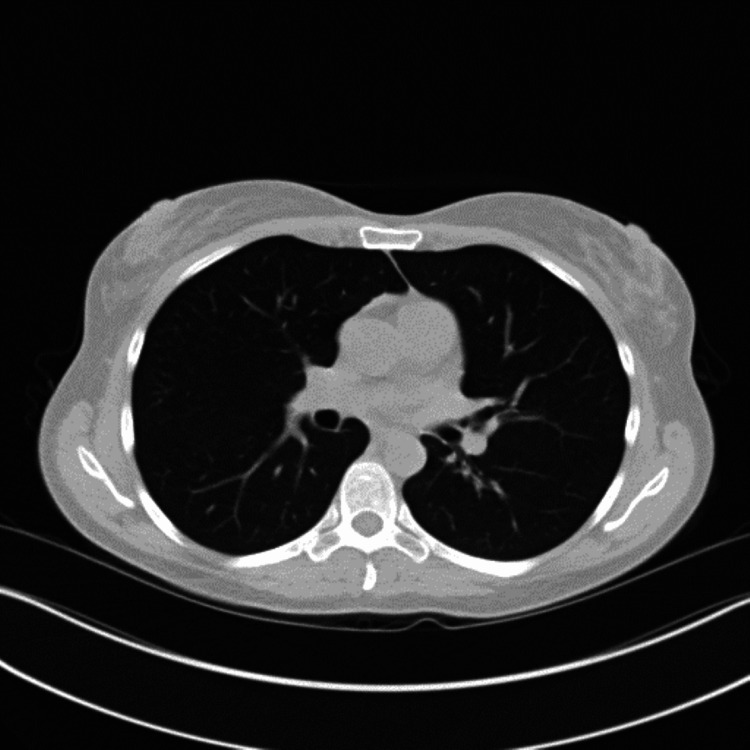
CT chest showing interval resolution of previously seen ground-glass opacities

Further immunological tests demonstrated blood immunoglobulin levels (IgG), which were found to be low (469 mg/dl), and the diagnosis of IgG deficiency was established. IgA (126 mg/dl) and IgM (112 mg/dl) were normal. Complete blood count with differential revealed absolute lymphocytes of 0.8 (0.8-5.3 10e3/uL) with 16% differential. Absolute CD19 B cells were 143 (107-698 cells/uL), and human immunodeficiency virus (HIV) was negative. She started weekly IgG replacement therapy (Gammaplex 10% and sourced from the USA), and there was immediate interval radiologic and clinical improvement. Prednisone was weaned off completely with sustained clinical and radiologic improvement (Figure 6). Repeat IgG levels normalized after several months on replacement therapy.

## Discussion

To our knowledge, this is one of the very few descriptions of SARS-CoV-2-associated OP treated successfully with IgG replacement. The exact cause of OP remains unknown, and it may be referred to as cryptogenic when there is no identifiable associated clinical condition or culprit [1,10]. In other clinical scenarios, it may be associated with secondary causes such as autoimmune disorders, viral infections, medications, radiotherapy, and toxic inhalations [2,5,9]. In our case, the cause of OP was thought to be due to SARS-CoV-2 given the history and timeline of the viral infection in relation to the diagnosis of organizing pneumonia. The duration following an acute COVID-19 infection of about three to six months has been identified in some studies as one of the main key factors in determining the prevalence of SARS-CoV-2-associated OP [11,12]. A study showed confirmatory lung biopsies performed six months after SARS-CoV-2 revealed OP as a possible complication following histological evaluation [13]. Another study stipulated that the probability of developing OP-like symptoms three months after hospitalization for confirmed COVID-19 infection was 40%, 25%, and 15% for critical, severe, and moderate patients, respectively [8]. Our patient was diagnosed with IgG deficiency following persistent OP recalcitrant to steroids and immunosuppressive agents. The exact etiology of IgG deficiency remains controversial. The causes can be divided into primary and secondary. Primary causes are thought to be related to genetic disorders such as X-linked agammaglobulinemia, hyper-IgM syndrome, etc. [14]. Secondary causes include malnutrition, medications, cancer, protein-losing nephropathy, traumatic injuries, and gastrointestinal losses [15]. From a broad perspective, primary causes are usually due to inadequate production of immunoglobulins, while secondary causes may be due to both increased loss and inadequate production.

Following SARS-CoV-2 pneumonitis, a subset of patients are left with recurrence or recalcitrant inflammatory lung disease [6]. The diagnosis of COVID-19-associated OP may be made in SARS-CoV-2-positive patients with classic symptoms and typical radiologic changes as described earlier. The definitive diagnosis usually requires microscopic and histological evaluation. The reported prevalence of SARS-CoV-2-associated OP in severe and critical cases is about 10-40% based on recent data [8,16]. Corticosteroids are the cornerstone of therapy for OP [2,4]. Relapses commonly occur when the corticosteroid dosing is being weaned down or following cessation [9]. There have been reports of corticosteroid-resistant and refractory cases of OP requiring alternative management options, including macrolides, cytotoxic therapy, and steroid-sparing immunosuppressants [17]. In our case, OP remained persistent even with prednisone doses above 20 mg in addition to mycophenolate 1000 mg twice daily. In a large cohort of critically ill COVID-19 patients, the incidence of IgG deficiency was common (21%), and IgG deficiency was associated with several clinical markers of increased disease severity, both at baseline and throughout the hospital course [18]. Vrettou and colleagues showed low IgG was an independent predictor for poor outcomes in a large prospective study [19]. Another study showed that patients with reduced total IgG were significantly more likely to require non-invasive mechanical ventilation and had an increased prevalence of persistent OP [7]. SARS-CoV-2 may infect lung epithelium and escalate pulmonary immune dysregulation in patients with COVID-19, causing a relative deficiency in immunity [7]. The severe form of COVID-19 has been attributed to a dysfunctional innate immune response, including a deficient type I interferon response complicated with an over-responsive adaptive immunity [20]. The clinical course of severe COVID-19 may be caused by a significantly reduced number of natural killer (NK) cells, while the systemic pro-inflammatory response to SARS-CoV-2 infection is regulated by complement activation [20]. A recent retrospective study on sixteen patients with a median COVID-19 duration of four weeks showed that treatment with IVIG was associated with clinical cure and viral clearance in immunocompromised patients [21]. Low IgG levels were found in our patient, and eventual improvement with IgG replacement therapy may have explained why OP was refractory to steroids and immunosuppressive therapy. Monthly IgG replacement therapy led to complete clinical and radiologic resolution without any dependence on steroids or immunosuppressant agents. The decision to treat is usually dependent on clinical history, trough serum IgG levels, history of recurrent infections, impaired response to vaccines, response to antibiotics, and persistent radiologic findings [15]. The normal ranges are variable and dependent on age. In adults, IgG levels of 300-600 mg/dl are indicative of mild reduction, and < 300 mg/dl is indicative of significant reduction [15]. Serum IgG levels > 600 mg/dl are usually the target for most prescribing medical providers when therapy is started. Intravenous IgG replacement therapy at dosages of 400 to 600 mg/kg should be given by infusion every four weeks [15].

The patient was seen back in the clinic with significant improvement in pulmonary function and was able to return to being active.

## Conclusions

IgG deficiency might be underdiagnosed in patients with SARS-CoV-2-associated OP and might serve as an indicator of increased disease severity and poor response to steroids as well as immunosuppressive agents. IgG replacement therapy may be an important adjunct therapy, and further prospective studies are needed to establish its applicability for this subset of COVID-19 patients. More studies are needed to better understand the relationship between SARS-CoV-2 and IgG deficiency.
